# Serum Antigenome Profiling Reveals Diagnostic Models for Rheumatoid Arthritis

**DOI:** 10.3389/fimmu.2022.884462

**Published:** 2022-04-20

**Authors:** Peng Han, Chao Hou, Xi Zheng, Lulu Cao, Xiaomeng Shi, Xiaohui Zhang, Hua Ye, Hudan Pan, Liang Liu, Tingting Li, Fanlei Hu, Zhanguo Li

**Affiliations:** ^1^ Department of Rheumatology and Immunology, Peking University People’s Hospital and Beijing Key Laboratory for Rheumatism Mechanism and Immune Diagnosis (BZ0135), Beijing, China; ^2^ Department of Biomedical Informatics, School of Basic Medical Sciences, Peking University, Beijing, China; ^3^ Peking-Tsinghua Center for Life Sciences, Peking University, Beijing, China; ^4^ State Key Laboratory of Natural and Biomimetic Drugs, School of Pharmaceutical Sciences, Peking University, Beijing, China; ^5^ State Key Laboratory of Dampness Syndrome of Chinese Medicine, The Second Affiliated Hospital of Guangzhou University of Chinese Medicine, Guangzhou, China; ^6^ Department of Integration of Chinese and Western Medicine, School of Basic Medical Sciences, Peking University, Beijing, China

**Keywords:** rheumatoid arthritis, antigenome, biomarkers, mass spectrometry, random forest

## Abstract

**Objective:**

The study aimed to investigate the serum antigenomic profiling in rheumatoid arthritis (RA) and determine potential diagnostic biomarkers using label-free proteomic technology implemented with machine-learning algorithm.

**Method:**

Serum antigens were captured from a cohort consisting of 60 RA patients (45 ACPA-positive RA patients and 15 ACPA-negative RA patients), together with sex- and age-matched 30 osteoarthritis (OA) patients and 30 healthy controls. Liquid chromatography-tandem mass spectrometry (LC-MS/MS) was then performed. The significantly upregulated and downregulated proteins with fold change > 1.5 (*p* < 0.05) were selected. Based on these differentially expressed proteins (DEPs), a machine learning model was trained and validated to classify RA, ACPA-positive RA, and ACPA-negative RA.

**Results:**

We identified 62, 71, and 49 DEPs in RA, ACPA-positive RA, and ACPA-negative RA, respectively, as compared to OA and healthy controls. Typical pathway enrichment and protein–protein interaction networks were shown among these DEPs. Three panels were constructed to classify RA, ACPA-positive RA, and ACPA-negative RA using random forest models algorithm based on the molecular signature of DEPs, whose area under curve (AUC) were calculated as 0.9949 (95% CI = 0.9792–1), 0.9913 (95% CI = 0.9653–1), and 1.0 (95% CI = 1–1).

**Conclusion:**

This study illustrated the serum auto-antigen profiling of RA. Among them, three panels of antigens were identified as diagnostic biomarkers to classify RA, ACPA-positive, and ACPA-negative RA patients.

## Introduction

Rheumatoid arthritis (RA) is a chronic autoimmune disease that leads to joint damage, systemic inflammation, and early mortality (1). The prevalence of RA was approximately 0.5%–1% worldwide and 0.28% in China (2, 3). The joint inflammation, combined with extra-articular complications, causes disability and reduces quality of life (4). Early diagnosis and subsequent treatment can substantially slow the progression of joint damage, thereby preventing irreversible disability (5).

Though the precise molecular mechanism in the triggering and progression of systemic immune response is not fully understood, the emergence of antibodies against self-antigens marks the loss of self-tolerance and can serve as a diagnostic biomarker (6). Among these are rheumatoid factor (RF) and anti-citrullinated protein antibodies (ACPAs), which are currently used as biomarkers for diagnostics, and other anti-modified protein antibodies (AMPAs) (7–9). The combination of autoantibody and self-antigen could form immune complexes that significantly augment the immune response and contribute to the inflammatory process of RA (10). Multiple antigens have been confirmed such as α-enolase, fibrinogen, filaggrin, vimentin, and type II collagen (11, 12). However, the profiling of serum antigen, antigenome, remains poorly known.

For decades, research has focused on single antigen identified as biomarkers (13). However, none of those achieves better specificity and sensitivity than ACPA alone. In this study, we broadened the focus by addressing the entire repertoire, aiming to capture the enormous biodiversity of antigens, with the goal to find a panel of diagnostic biomarkers instead of a single candidate. Moreover, the approach allows for finding differences of immune response by clustering the antigen repertoire that share certain function and pathway, providing further evidence in understanding of RA pathophysiology.

The robust growth of quantitative proteomic methods enables researchers to discover indicator proteins for diagnosis and treatment of diseases. There has been a recent expansion in proteomics research on a number of different rheumatic diseases (14–16). Due to the large datasets generated by proteomics, it requires informatic approaches such as machine learning techniques to analyze and interpret data, which have been exploited to predict biomarkers to accurate classify different diseases (17–19). We employed a robust mass spectrometry (MS)-based proteomics strategy to delineate the serum antigenomic profiling. By applying a widely used machine-learning algorithm, random forest, we described 3 panels of biomarkers to distinguish RA, ACPA-positive RA, and ACPA-negative RA. These biomarkers were further validated in a cohort using proteomic data. These findings provided knowledge about serum antigen in RA and might reveal potential therapeutic targets.

## Materials and Methods

### Study Population and Serum Sample Collection

Serum from 60 RA patients, as well as sex- and age-matched 30 osteoarthritis (OA) patients and 30 healthy controls were collected at the Department of Rheumatology and Immunology, Peking University People’s Hospital, Beijing, China. The study was approved by the Research Ethics Committee of Peking University People’s Hospital. Informed consent was obtained from all patients and healthy donors. The study population was randomly split into a test cohort (36 RA, 18 OA, and 18 HC) and a validation cohort (24 RA, 12 OA, and 12 HC). Detailed clinical and demographic characteristics are summarized in **Table 1**.

**Table 1:** Clinical and laboratory characteristics of RA patients and controls in the study.

Characteristics	RA (*n* = 60)	OA (*n* = 30)	HC (*n* = 30)
Age, mean (range), years	61.77 (44–78)	64.27 (46–81)	62.37 (52–69)
Gender, no. male/female	11/49	7/23	8/22
Duration, mean (range), years	12.37 (1–42)	–	–
ESR, mean (range), mm/h	39.28 (5–106)	–	–
CRP, median (range), mg/L	23.85 (0.22–172)	–	-
RF, median (range), IU/ml	327.4 (2–3750)	–	–
Anti-CCP, median (range), U/ml	147.4 (1.93–296.9)	–	–
WBC, median (range), 10^9^/L	5.793 (2.6–12.3)	–	-
TJC, median (range)	7 (0–22)	–	-
SJC, median (range)	5 (0–21)	–	-
DAS28, median (range)	4.258 (1.15–6.93)	–	-
Medication, no (%)			
Steroids	25 (41.67%)	–	–
NSAIDs	13 (21.67%)	–	–
DMARDs	59 (98.3%)	–	–
Biologics	31 (51.67%)	–	–

HC, healthy controls; ESR, erythrocyte sedimentation rate; CRP, C-reactive protein; RF, rheumatoid factor; Anti-CCP, anti-cyclic citrullinated peptide antibody; WBC, white blood cell; TJC, tender joint count; SJC, swollen joint count; DAS28, disease activity score 28; NSAIDs, nonsteroidal anti-inflammatory drugs; DMARDs, disease-modifying anti-rheumatic drugs.

All RA patients met the 2010 American College of Rheumatology (ACR)/European League Against Rheumatism (EULAR) classification criteria (20). The exclusion criteria include active infection, malignancy, and other known autoimmune or immune-mediated diseases, such as systemic lupus erythematosus, Sjogren’s syndrome, and type I diabetes. The individuals (*n* = 10 in each group) selected for IgG purification were required to be free from monoclonal antibody treatment in at least 6 months.

### Sample Preparation and Tryptic Digestion

IgG from human serum was purified using protein G spin kit (Catalog No.22852, Thermo Fisher Scientific). IgG was purified from 500 μl of pooled serum of ten patients according to the manufacturer’s instrument, representing the repertoire of antibodies of each group. The eluted IgG was then washed and concentrated using 30-kDa MWCO filters (Catalog No. UFC803096, Amicon, Millipore). To capture serum antigen, 5 mg of IgG was coupled to 1 ml of CNBr-activated Sepharose 4B column (Catalog No.17043001, GE). By pretreating the IgG column with acidic elution buffer (10 mmol/L Gly-HCl, pH = 2.8), the antigens bound to IgG were eluted. Then, the diluted serum of one patient was incubated at room temperature with end-over-end mixing for 1 h. Bound antigens were eluted with acidic elution buffer (10 mmol/L Gly-HCl, pH = 2.8) and immediately neutralized by Tris-HCl (1 mmol/L, pH = 9.1). The concentration of the protein was determined by Bradford protein assay (Catalog No. DQ101-01, Transgen Biotech) and then stored at −80°C .

### LC-MS/MS and Data Analysis

Protein (10 μg) was hydrolyzed with trypsin. Digested products were separated by a 120-min gradient elution at a flow rate of 0.300 µL/min with the Thermo Ultimate 3000 nano-UPLC system, which was directly interfaced with the Thermo Fusion LUMOS mass spectrometer. The analytical column was an Acclaim PepMap RSLC column (75 µm ID, 250 mm length, C18). Mobile phase A consisted of 0.1% formic acid, and mobile phase B consisted of 100% acetonitrile and 0.1% formic acid. The single full-scan mass spectra were acquired in a data-dependent manner in the Orbitrap at a mass resolution of 60,000 at 375–1500 m/z. Xcalibur 4.1.50 software was used for data acquisition. Protein identification was carried out using Mascot and Sequest search algorithms through the Proteome Discovery software (version 2.4). Searches were carried against Human RefSeq protein database. MS tolerance was set to 10 ppm while MS/MS tolerance was set to 0.02 Da. The peptide-spectrum match allowed 1% target false discovery rate (strict). We used label-free quantification (LFQ) algorithm to quantify protein expression and peptide-spectrum matching. Normalization was performed against the total peptide amount. Immunoglobulins and post-translational modifications are not analyzed in the study, but could be potentially analyzed in the future.

### Bioinformatic Analysis

To obtain the intersection of antigen among RA, OA, and healthy controls, we used the Venn diagram software (http://bioinformatics.psb.ugent.be/webtools/Venn/). Pathway enrichment analysis was performed to classify proteins based on molecular function and biological processes by Metascape web-based platform (21). Protein–protein interaction of differentially expressed proteins was performed using Search tool for the retrieval of interaction gene/protein (STRING) database (PPI enrichment *p*-value < 1.0e-16) and visualized by Cytoscape plug-in Cytohubba (22, 23).

### Statistical Analysis and Machine Learning

Missing values were imputed with the minimal values for each feature. To get differentially expressed proteins, the fold change and *t*-test *p*-value were calculated between RA, ACPA-positive RA, ACPA-negative RA, and control (OA and healthy controls). The protein whose *p*-value < 0.05 and fold change > 1.5 was defined as differentially expressed protein. The heatmaps were drawn using the R package “pheatmap” (version 1.0.12), the sum of *z*-scores of log-transformed values were displayed, and the rows were sorted by fold changes. The PCA was performed using the function “decomposition.PCA” in scikit-learn (version 0.23.1) with default parameters. The log-transformed values were used as input for PCA.

The random forest classifiers were build using the function “ensemble.RandomForestClassifier” in scikit-learn (version 0.23.1), with 101 trees, and the max depth for the trees was set to 4 to avoid overfitting. The log-transformed values of differentially expressed proteins were used as input features, and the number of features to consider in each tree was sqrt (number of features). We deleted SAA (D3DQX7) as the sequence was very similar to SAA1 and SAA2. The importance of proteins was calculated using the build-in function “feature_importances_”, which provides the impurity-based feature importance. The train-test split and classification process were repeated 500 times to calculate the AUC and feature importance.

## Results

### Patients and Study Design

We procured a cohort of patients containing 60 RA, 30 OA, and 30 healthy controls. The detailed clinical and demographic characteristics are shown in **Table 1**. The median age was 61.77 years and 81.7% of the patients were female. Thirty OA patients and 30 healthy controls were all age- and sex-matched. The disease duration ranged from 1 to 42 years, with a mean duration of 12.37 years. Seventy-five percent (45 of 60) of RA patients were ACPA-positive. The mean ESR (erythrocyte sedimentation rate) and CRP (C-reactive protein) were 39.28 mm/h and 23.85 mg/dl, respectively. The mean DAS28 score (Disease Activity Score-28) was 4.258.

**Figure 1:**
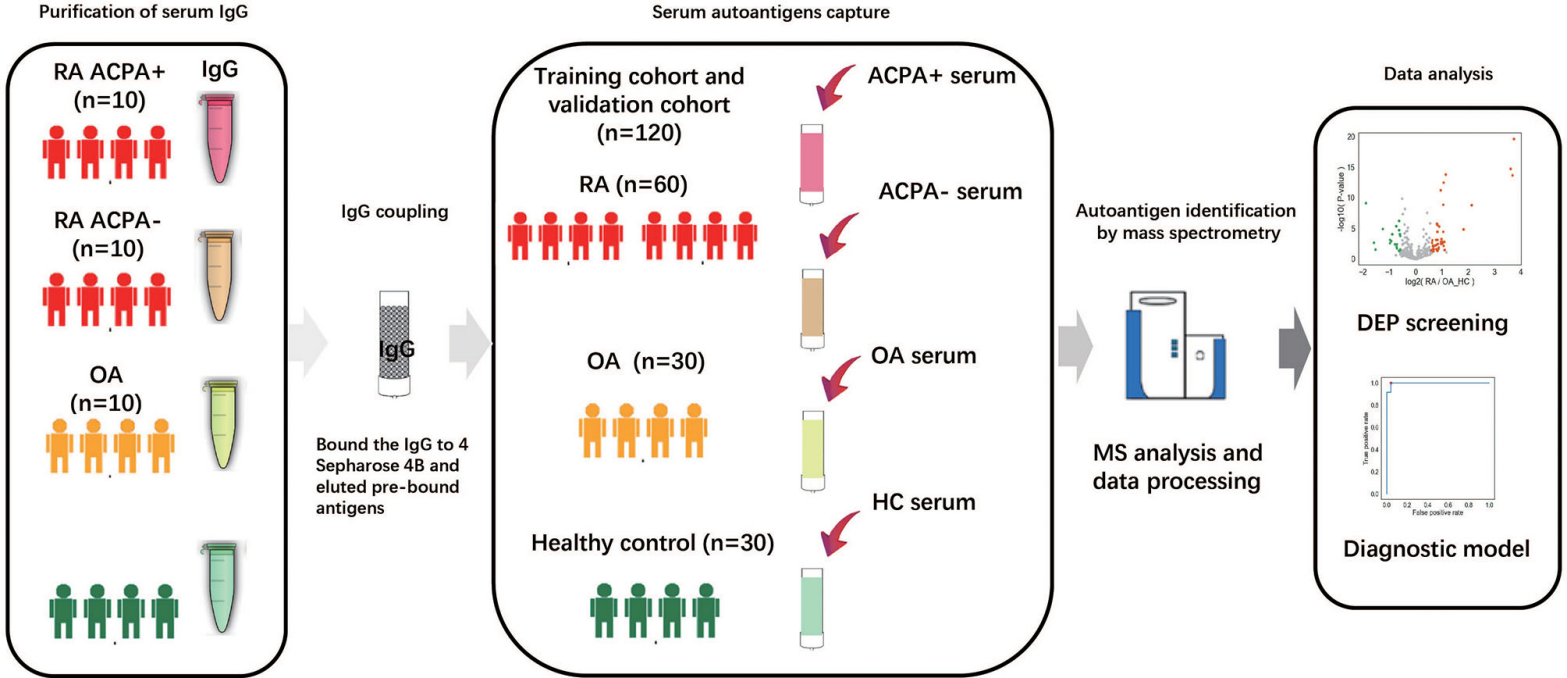
Study overview and antigenome characterization. Overview of the study cohort and schematic workflow. RA, rheumatoid arthritis; OA, osteoarthritis; ACPA, anti-citrullinated protein antibody; HC, healthy control; MS, mass spectrometry; DEP, differentially expressed protein.

The workflow employed for this study is shown in **Figure 1**. Briefly, IgGs were purified from 500 μl of pooling mixture serum of 10 individuals in each group, respectively. These IgGs were bound to the Protein G column and then were treated to remove the antigens potentially bound to the antibodies first. After that, serum antigens from 120 samples were purified and collected individually. The antigen peptide mixture of each sample was then analyzed and quantified by high-resolution liquid chromatography with tandem mass spectrometry (LC-MS/MS) (24).

### Serum Antigenomic Profiling of RA Patients

**Figure 2:**
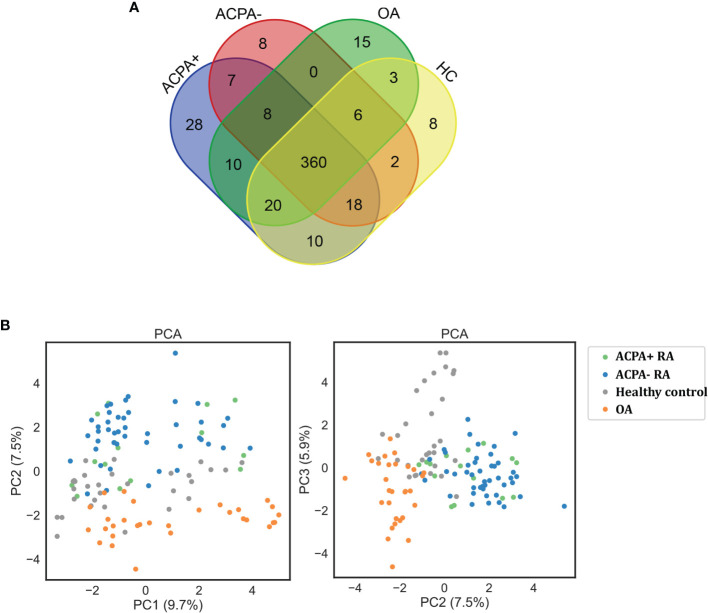
Protein quantification through LC-MS/MS. **(A)** Venn diagram of the identified proteins among RA patients and controls. **(B)** Clustering analysis of differentially expressed proteins on PCA analysis. ACPA+, ACPA-positive RA; ACPA-, ACPA-negative RA; PCA, principal component analysis.

Applying this workflow, we quantified 4,475 proteins and 12,217 peptides from 120 samples. With immunoglobulins excluded, 461 proteins in ACPA-positive RA, 409 proteins in ACPA-negative RA, 427 proteins in OA patients, and 422 proteins in healthy control were identified. A total of 360 proteins were in common among the 3 groups, while 35 proteins were specific for ACPA-positive RA and 15 for ACPA-negative RA. Eight proteins were found only in the ACPA-negative group and 28 in the ACPA-positive group (**Figure 2A**). Proteins with high confidence and could be detected in more than 20% in a particular patient group were chosen for further analyses.

The principal component analysis (PCA) showed that the clustering of samples is clearly classified into different groups as RA, OA, and healthy controls (**Figure 2B**). However, PCA analysis could not distinguish ACPA-negative RA from ACPA-positive RA patients, demonstrating their similar antigenome pattern. Taken together, these data presented a deep antigenome coverage, a promising basis for discovery of biomarkers.

### Analysis of Differentially Expressed Proteins

**Figure 3:**
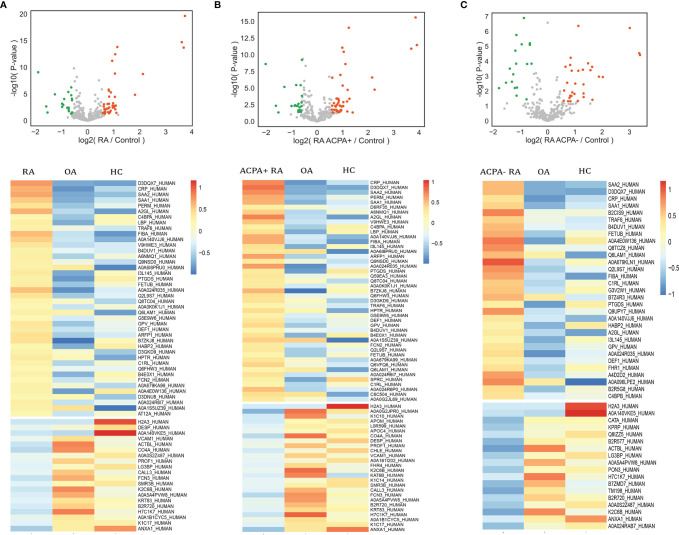
Analysis of differential expressed proteins. Volcano plots compare RA **(A)**, ACPA-positive RA **(B)**, ACPA-negative RA **(C)**, and controls. Heatmap analysis of proteins that differ significantly (*p* < 0.05, fold change > 1.5) in abundance in RA **(A)**, ACPA-positive RA **(B)**, and ACPA-negative RA **(C)**.

We next assessed significant quantitative differences between RA, OA, and healthy controls. We selected significantly upregulated and downregulated proteins by >1.5-fold (*p* < 0.05). A total of 62 differentially expressed proteins (DEPs) such as fibrinogen alpha chain, lipopolysaccharide-binding protein, and serum amyloid protein in RA were identified and shown in the volcano plot (**Figure 3A**). Heatmap analysis was performed to visualize those proteins (**Figure 3A**). We next found 71 proteins differentially expressed in ACPA-positive and 49 proteins differentially expressed in ACPA-negative patients, using the same filter criteria (**Figures 3B, C**).

**Figure 4:**
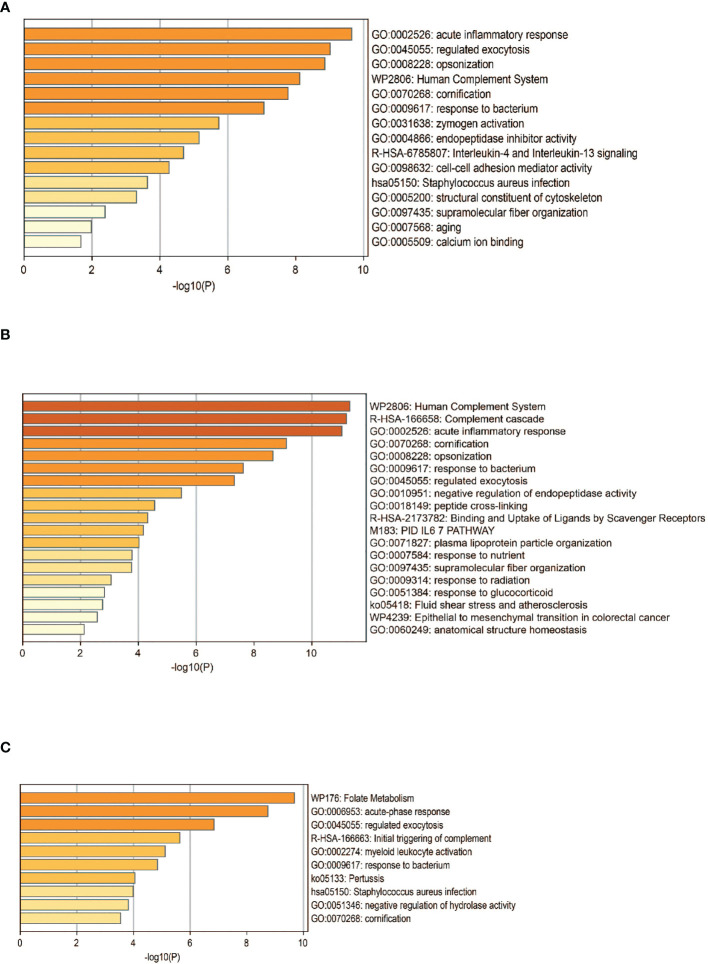
Functional analysis of DEPS. Pathway analysis of DEPs in patients with RA **(A)**, ACPA-positive RA **(B)**, and ACPA-negative RA **(C)**. GO, gene ontology.

The DEPs were then subjected to enrichment analysis (**Figure 4**). The analysis revealed that DEPs of these 3 groups were significantly enriched in pathways associated with immunology and inflammatory response, “acute inflammatory response”, “activation of complement system”, and “humoral immune response”. DEPs in RA were enriched in pathways including “cell-cell adhesion” and “IL-4 and IL-13 signaling”. The pathways of DEPs in ACPA-positive RA were enriched in processes involved in “binding and uptake of ligand of scavenger receptors” and “IL-6 pathway”. Some pathways associated with metabolic process were enriched in DEPs of ACPA-negative RA, such as “folate metabolism”, which might be interesting in future studies.

**Figure 5:**
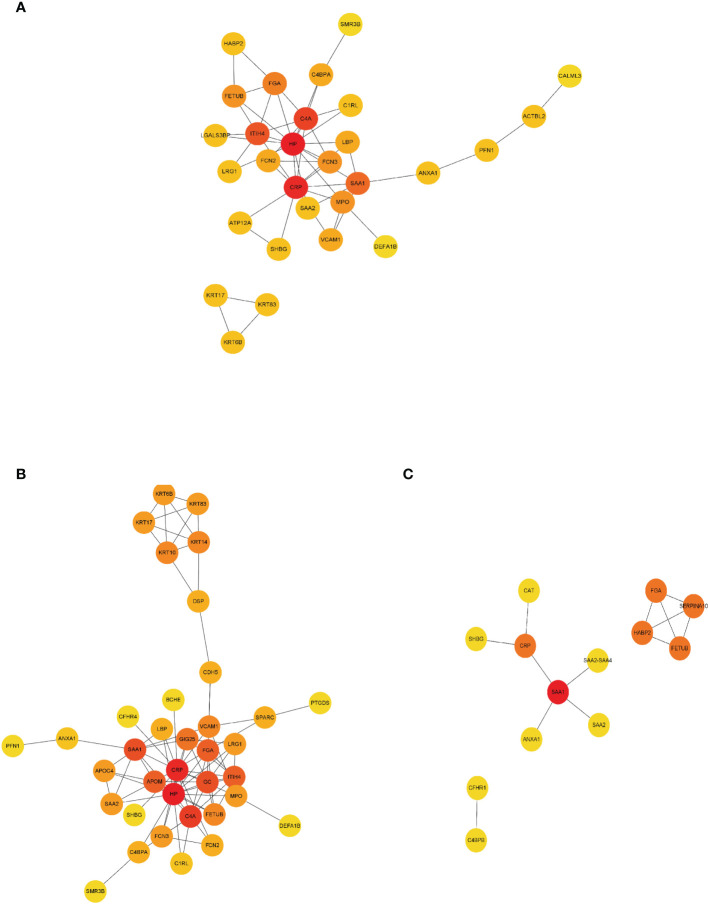
PPI network construction of DEPs. Interaction network analysis of DEPs in RA **(A)**, ACPA-positive RA **(B)**, and ACPA-negative RA **(C)** by STRING and Cytoscape. Cytohubba plug-in was applied to identify the hub proteins in the network by protein degrees. Red indicated DEPs were at the center of the network and possessing 5–10 edges. Orange indicated DEPs possessing 3–5 edges. Yellow indicated DEPs possessing 1 to 2 edges. PPI, protein–protein interaction.

As shown by protein–protein interaction (PPI) analysis of DEPs by the STRING database, the antigenome possessed abundant interactions. To recognize the key antigens lying in an essential position, we exploited Cytoscape plugin Cytohubba, which identified hub proteins in the networks. As shown in **Figure 5**, DEPs such as haptoglobin and ITIH4 were screened out as top hub proteins based on the connectivity degree. Both haptoglobin and ITIH4 could function as acute-phase reactants (25, 26). It was previously reported that the levels of haptoglobin were elevated in RA serum (27). ITIH4 was found to be a serum biomarker for a variety of malignancies including gastric cancer and hepatocellular carcinoma (28, 29). However, there is limited research investigating their detailed role in RA. These proteins might be essential in the pathogenesis of RA and utilized as biomarkers after rigorous validation.

### Machine Learning for Identification of RA Patients

Next, we attempted to discriminate RA from OA and healthy controls based on the DEPs. A widely used machine learning algorithm, random forest, was used to classify the patients. The model was trained on 60% of the samples (36 RA, 18 OA, and 18 healthy controls) and evaluated on the remaining 40% of the samples (24 RA, 12 OA, and 12 healthy controls). We repeated this process 500 times to calculate the area under the curve (AUC) of the receiver operating characteristic curve and feature importance for each antigen.

**Figure 6:**
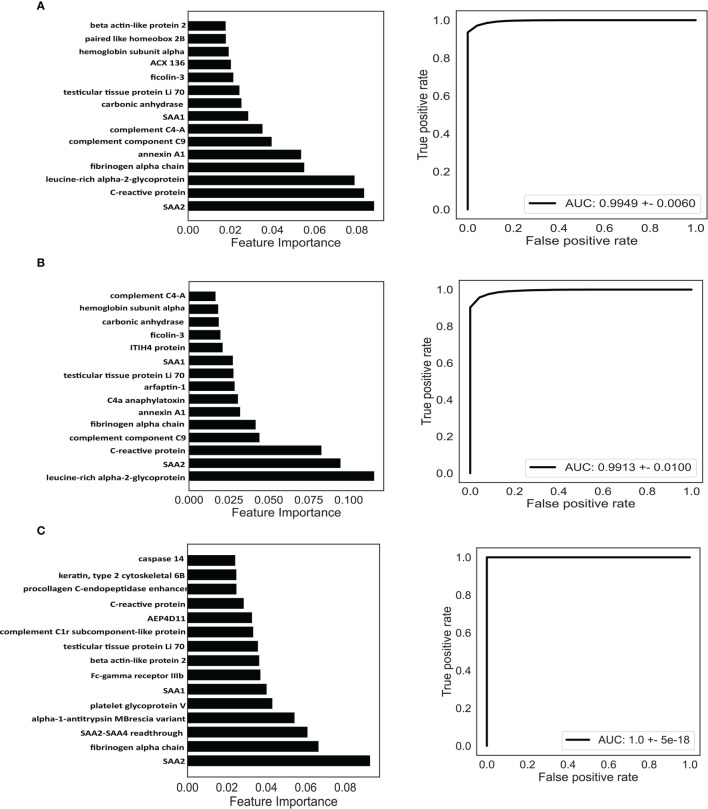
Identification of potential biomarkers based on machine learning. Classification of RA **(A)**, ACPA-positive RA **(B)**, and ACPA-negative RA **(C)**. Top 15 proteins prioritized by random forest analysis (left). ROC of the random forest model in the test cohort (right). AUC, area under curve.

For the classification of RA, the AUC of the random forest model reached 0.9949 (95% confidence interval [CI] = 0.9792–1) (**Figure 6A**), and the top 15 dominant antigens in the model were SAA2, C-reactive protein (CRP), leucine-rich alpha-2-glycoprotein, fibrinogen alpha chain, annexin A1, complement component C9, complement C4-A, SAA1, carbonic anhydrase, testicular tissue protein Li 70, ficolin-3, ACX136, hemoglobin subunit alpha, paired like homeobox 2B, and beta-actin-like protein 2 (**Figure 6A**).

Next, we investigated the possibility of discriminating ACPA-positive and ACPA-negative RA patients from OA patients and healthy controls based on the DEPs. Random forest algorithm was employed as well; 60% of the samples were used to train and 40% of the samples were used to evaluate. For ACPA-positive RA patients, the model reached an AUC of 0.9913 (95% CI = 0.9653–1) (**Figure 6B**), and the top 15 best-performing proteins were leucine-rich alpha-2-glycoprotein, SAA2, CRP, complement component C9, fibrinogen alpha chain, annexin A1, C4a anaphylatoxin, arfaptin-1, testicular tissue protein Li 70, SAA1, ITIH4 protein, ficolin-3, carbonic anhydrase, hemoglobin subunit alpha, and complement C4-A (**Figure 6B**). Moreover, for the classification of ACPA-negative RA patients, the AUC was calculated as 1.0 (95% CI = 1–1) (**Figure 6C**). The top 15 proteins were SAA2, fibrinogen alpha chain, SAA2-SAA4 readthrough, alpha-1-antitrypsin MBrescia variant, platelet glycoprotein V, SAA1, Fc-gamma receptor IIIb, beta-actin-like protein 2, testicular tissue protein Li 70, complement C1r subcomponent-like protein, AEP4D11, CRP, procollagen C-endopeptidase enhancer, Keratin type II cytoskeletal 6B, and caspase 14 (**Figure 6C**).

## Discussion

Our study presented a serum antigenomic investigation of RA using label-free global proteome strategy, which offered a landscape view of antigens. Using random forest, an ensemble, supervised machine learning algorithm, 3 diagnostic signatures were built to classify RA, ACPA-positive RA, and ACPA-negative RA patients. Our findings might help to understand the pathogenesis of RA and provide novel and specific diagnostic targets for the disease.

Early diagnosis and immediate, effective therapy are crucial to gain control of inflammation and prevent deterioration, functional disability, and unfavorable progression in RA patients. To carry out personalized medicine for RA, clinical practice requires the use of biomarkers to ensure diagnosis, accurate stratification, and the high efficacy of treatment. Current clinically used biomarkers including anti-CCP and RF only shows a modest discriminating power due to the lack of sensitivity and specificity (30). Searching biomarkers for diagnosis is a continuous effort, but none of those translate into routine clinical use (31). Therefore, searching reliable biomarkers for RA in a large population is highly desirable. To address the problem, we have combined cutting-edge mass spectrometry hardware, MS data processing, and bioinformatic analysis to build a high-performance serum antigenomic workflow.

Other groups have also performed proteomic studies for RA. Mun et al. performed a quantitative proteomic study and identified 5 biomarkers using RA serum, which were quantitively verified by multiple reaction monitoring (MRM) (32). Colasanti et al. discovered that anti-Hcy-A1AT (homocysteinylated alpha 1 antitrysin) autoantibody could be considered as a potential biomarker for RA by using matrix-assisted laser desorption/ionization-time of flight (MALDI-TOF/TOF) (33). However, we used a novel approach to capture the set of serum antigens, which was advantageous as it focused on a more targeted set of proteins, compared to entire serum proteome. We identified confirmed and putative antigens as candidates of novel potential biomarkers. In total, 4,475 proteins were identified by label-free comparative proteomic analysis of antigen profiling of RA, OA, and healthy control serum. Compared to OA and healthy controls, we found 62 DEPs (FC > 1.5, *p* < 0.05) in RA, 71 DEPs that were specific in ACPA-positive patients, and 49 DEPs specific in ACPA-negative patients. We tried to gain insight into the functional roles of these DEPs associated with RA *via* pathway enrichment analysis. The interaction of DEPs was shown based on PPI networks. Moreover, we identified hub proteins in the interaction networks. These avenues of enquiry may provide insight into the underlying mechanisms of RA.

As a single biomarker may hardly achieve satisfactory discriminating power, seeking multiple biomarkers and developing a combinatorial model is a compromising strategy. By virtue of comprehensive antigenome profiling and random forest algorithm, we revealed 3 predictive models for RA, ACPA-positive RA, and ACPA-negative RA. The models have achieved low classification errors and resulted in very high AUC levels.

Compared to ACPA-positive RA, ACPA-negative RA is poorly understood in etiology and pathogenesis. Lack of effective biomarkers impedes early diagnosis and treatment, highlighting the importance of identifying specific antigens in this subset (34). It is worth noting that several antigens are unique in the ACPA-negative model, such as SAA2-SAA4 readthrough, platelet glycoprotein V, Fc-gamma receptor IIIb, complement C1r subcomponent-like protein, procollagen C-endopeptidase enhancer, and caspase 14 (**Figure 6C**). These proteins participate in various biological processes including acute phase response, cornification, complement activation, innate immune response, platelet activation, and collagen binding (35, 36). There is limited research investigating their function and association with autoimmune or inflammatory diseases (37). Validation by ELISA in larger ACPA-negative RA cohorts and exploration of their detailed functions in the disease are needed. Hopefully, these newly identified antigens may help early diagnosis and hint underlying mechanism of ACPA-negative RA.

Though assay results were promising, this study does have limitations. First, we utilized IgG purified from ACPA-positive patients to capture antigens rather than anti-CCP2 antibody, which resulted in an incomplete reactivity pattern of ACPA-IgG due to its lower affinity toward citrullinated antigens. Besides, by using an IgG column to capture serum antigens from the corresponding group of patients, the DEPs identified between groups might be partially due to the differences of IgG binding ability from various patients rather than antigens that were purely from the capture. Second, if the model is to be applied in clinic, more rigorous quantification and extensive validation by targeted protein quantification or ELISA are still needed. The detailed roles of antigens in the pathogenesis of RA should be further elucidated or experimentally validated. Third, this was a single-center study, and the results merit validation in a larger, multicenter study that involve OA, systemic lupus erythematosus (SLE), ankylosing spondylitis (AS), psoriatic arthritis (PsA), gout patients, etc. Lastly, this work establishes the foundation for longitudinal studies geared toward the development of models predictive of disease onset or progression, and efficacy after treatment. The sera samples were collected at a single time point in both RA patients and control, and future studies of sera from more time points along the disease course are required, which could be potentially utilized to explore molecular dynamics during disease progression.

In summary, we employed label-free global proteomics technology to analyze serum antigenome profiling of RA. The study increased our understanding of RA antigens and identified potential biomarkers to provide novel and specific diagnostic targets for the disease. We suggest that these panels identified here could be utilized as multiplex protein microarray platforms that have potential for scalability and contribute toward improved decision-making.

## Data Availability Statement

The datasets presented in this study can be found in online repositories. The names of the repository/repositories and accession number(s) can be found at: http://www.proteomexchange.org/, PXD031498.

## Ethics Statement

The studies involving human participants were reviewed and approved by the Research Ethics Committee at Peking University of People’s Hospital. The patients/participants provided their written informed consent to participate in this study.

## Author Contributions

FH and ZL designed the research. PH, XZ, and LC collected blood sample and PH summarized clinical data. PH, XS, and XHZ performed LC-MS/MS. PH, CH, XS, XHZ, XZ, LC, TL, and FH analyzed and interpreted the data. CH and TL performed machine learning. PH and CH wrote the manuscript. HY, FH, HP, LL and ZL edited the manuscript. All authors contributed to the article and approved the submitted version.

## Funding

This work was supported by grants from Guangdong Basic and Applied Basic Research Foundation (2020B1515130005 to LL), the National Natural Science Foundation of China (U1903210 to ZL, 82171773 and 81971523 to FH), the Beijing Nova Program (Z181100006218044 and Z211100002121163 to FH), the Beijing Science and Technology Planning Project (Z191100006619109 to HY), Macao Science and Technology Development Fund (0094/ 2018/A3 to ZL), as well as by the Fundamental Research Funds for the Central Universities: Peking University Clinical Medicine Plus X-Young Scholars Project (PKU2021LCXQ014 to FH) and the Peking University People’s Hospital Research and Development Funds (RDX2020-01 to FH).

## Conflict of Interest

The authors declare that the research was conducted in the absence of any commercial or financial relationships that could be construed as a potential conflict of interest.

## Publisher’s Note

All claims expressed in this article are solely those of the authors and do not necessarily represent those of their affiliated organizations, or those of the publisher, the editors and the reviewers. Any product that may be evaluated in this article, or claim that may be made by its manufacturer, is not guaranteed or endorsed by the publisher.
